# Type distribution of human papillomaviruses in ThinPrep cytology samples and HPV16/18 E6 gene variations in FFPE cervical cancer specimens in Fars province, Iran

**DOI:** 10.1186/s12935-023-03011-8

**Published:** 2023-08-11

**Authors:** Ali Farhadi, Haniyeh Abuei, Mohammad Ali Okhovat, Bita Geramizadeh, Abbas Behzad-Behbahani, Pei Pei Chong, Negin Nikouyan, Sepide Namdari

**Affiliations:** 1 Diagnostic Laboratory Sciences and Technology Research Center, School of Paramedical Sciences, Shiraz University of Medical Sciences, Shiraz, Iran; 2 Department of Medical Laboratory Sciences, School of Paramedical Sciences, Shiraz University of Medical Sciences, Shiraz, Iran; 3 Department of Pathology, Medical School of Shiraz University, Shiraz University of Medical Sciences, Shiraz, Iran; 4 Transplant Research Center, Shiraz University of Medical Sciences, Shiraz, Iran; 5 School of Biosciences, Faculty of Health and Medical Sciences, Taylor’s University, Subang Jaya, Malaysia

**Keywords:** HPV, ThinPrep, Phylogenetic analysis, Lineage, HPV16, HPV18, Cervical cancer

## Abstract

**Background:**

There exists strong evidence that human papillomavirus (HPV) is associated with cervical cancer (CC). HPV E6 is a major oncogene whose sequence variations may be associated with the development of CC. There is not sufficient data on the distribution of HPV types in ThinPrep cytology specimens and HPV 16/18 E6 gene variations among CC patients in the southwest of Iran. This study was conducted to contribute to HPV screening and vaccination in Iran.

**Methods:**

A total of 648 women screened for cervicitis, intraepithelial neoplasia or CC were included in the study. All participants underwent ThinPrep cytology testing, single-step HPV DNA detection and allele-specific reverse hybridization assays. Moreover, a total of 96 specimens previously tested positive for single infection with HPV16 or 18 were included for variant analysis. HPV16/18 lineages and sublineages were determined by PCR assays followed by sequencing the E6 gene and the construction of neighbor-joining phylogenetic trees.

**Results:**

Overall, HPV DNA was detected in 62.19% of all the screened subjects. The detection rates of HPV DNA among individuals with normal, ASC-US, ASC-H, LSIL, and HSIL cervical cytology were 48.9%, 93.6%, 100%, 100%, and 100%, respectively. Low-risk HPVs were detected more frequently (46.9%) than high-risk (38.9%) and possible high-risk types (11.1%). Of 403 HPV-positive subjects, 172 (42.7%) had single HPV infections while the remaining 231 (57.3%) were infected with multiple types of HPV. Our results indicated a remarkable growth of high-risk HPV66 and 68 and low-risk HPV81 which have rarely been reported in Iran and HPV90 and 87 that are reported for the first time in the country. In addition, 3 lineages (A, D, and C) and 6 sublineages (A1, A2, A4, C1, D1, and D2) of HPV16, and one lineage and 4 sublineages (A1, A3, A4, and A5) of HPV18 were identified. The studied HPV16 and 18 variants mainly belonged to the D1 and A4 sublineages, respectively.

**Conclusion:**

The present study suggests that the prevalence of HPV infection in women of all age groups with or without premalignant lesions in the southwestern Iran is high and the predominant HPV types in the southwest of Iran may differ from those detected in other parts of the country. This study also highlights the necessity of not only initiating HPV vaccination for the general population but also developing new vaccines that confer immunity against the prevalent HPV types in the area and national cervical screening programs using a combination of thinPrep cytology test and HPV detection assays in order to improve the accuracy of the screening.

**Supplementary Information:**

The online version contains supplementary material available at 10.1186/s12935-023-03011-8.

## Background

Known as the fourth most common gynecological malignancy, cervical cancer (CC) was reported to account for about 604,000 new cases and approximately 342,000 deaths across the world in the year 2020 [1]. The incidence rate of CC is increasing in Iran and has been estimated to be 4.5 per 100,000 women [2]. Annually, one out of every 123 women develops CC and nine out of every 100,000 women die of this disease [3]. Persistent human papillomavirus (HPV) infection is known to be the primary cause of CC as well as the preceding premalignant lesions referred to as cervical intraepithelial neoplasia (CIN) [4]. The development of CC from CIN takes place through a long reversible process. Timely diagnosis and intervention during this process can reduce the risk of CC occurrence [5]. There exist 51 recognized HPV types that are classified into three distinct risk groups based on their association with CC. High-risk HPV (HR-HPV) types (16, 18, 31, 33, 35, 39, 45, 51, 52, 56, 58, 59, 66, and 68) are known to have great carcinogenic potential. Possible high-risk (pHR-HPV) types (26, 53, 67, 70, 73, and 82) are suspected of carcinogenesis but whether they are indeed associated with CC or not is yet to be proved. Low-risk HPV (LR-HPV) types (6, 7, 11, 13, 30, 32, 34, 40, 42, 43, 44, 54, 61, 62, 69, 71, 72, 74, 81, 83, 84, 85, 86, 87, 89, 90, 91, 97, 102, 106, and 114), on the other hand, are generally responsible for non-cancerous genital lesions [6–8].

Proper management of CC relies on early diagnosis and effective prophylactic vaccination [9]. There are HPV vaccines, such as GARDASIL^®^9, that target most of the HR-HPVs. However, not only has not HPV vaccination been included in the national vaccination program in Iran, but also the vaccines that are available in the market are only protective against HPV16 and 18. Furthermore, since the prevalence of HPV types varies by geographic region [10, 11], the distribution pattern of HPV types should be considered to select the most effective HPV vaccine for different populations as well as different age groups [12].

HPV types are classified into different lineages provided that there is a difference of 1%-10% in complete genomic nucleotide sequences, and further into different sublineages in case of 0.5%-1% sequence variation, allowing better identification of viral heterogeneity [13]. HPV16 variants have been classified into four principal lineages based on sequences published by Burk et al. [13], and also presented in the Papillomavirus Episteme database (http://pave.niaid.nih.gov). These lineages are as follows; lineage A, including sublineages A1–A3 (previously known as European variants) and A4 (previously known as Asian variant); lineage B, comprising sublineages B1, B2 (formerly known as African-1 variants), B3, and B4; lineage C, with sublineage C1 (formerly African-2 variant), C2, C3, and C4; and lineage D, consisting of sublineages D1 (previously North American variant), D2, D3 (Asian–American variants), and D4. Moreover, HPV18 variants have been classified into three main lineages: lineage A, including sublineages A1, A2 (previously known as Asian Amerindian variants), and A3–A5 (formerly known as European variants); lineage B, with sub-lineages B1–B3, and lineage C with the only sub-lineage named C1 (all previously known as African variants) [13]. According to epidemiological findings, HPV16 variants pose the risk of persistent infection, progression to pre-cancer, and cancer. Furthermore, HPV16 sublineages A3, A4, and D have been reported to be associated with higher risks of CC [14–16]. Conversely, most studies do not support any of HPV18 lineages or sublineages carrying a higher risk of cancer compared to others [17, 18]. However, these findings were not replicated globally and the inconsistent results highlight the necessity of further studies on the distribution of HPV16/18 lineages and sublineages in different regions and their oncogenicity considering ethnicity.

Undoubtedly, regional data on the prevalence and type distribution of HPVs are of great importance to evaluate the potential of currently available HPV vaccines to prevent CC. In addition, the knowledge of HPV type distribution in each country is pivotal to vaccine development and national vaccination programming. Although there are many reports on the distribution of different HPV types and variants worldwide, there seem to be few regional studies investigating the distribution pattern of HPV types [19, 20] and HPV16/18 variants in the Iranian population [21–24]. Previous studies in Iran have reported the prevalence of HPV in different cervical specimens to range from 5.5% to 9.4% in normal cytology specimens [25–27]; between 61.7% and 65.3% in CIN I–III samples [26, 28, 29], and between 75.2% and 87% in CC specimens [30–32]. However, regardless of cervical cytology result, the overall prevalence of HPV infection was reported to be 52.25% in female outpatients referred to the laboratories of Tehran, the capital of Iran, between 2019 and 2021. The rate of HR-HPV and LR-HPV types among these patients were 42.1% and 57.9%, respectively [33]. Herein, we have examined the type-specific prevalence of HPVs with a large sample size and further investigated the E6 gene-based genetic variability of HPV16/18 lineages and sublineages according to the severity of the cervical lesions in the southwest of Iran.

## Methods

### Study subjects

A total of 722 women who attended the outpatient office of Shahid Motahari Gynecology Clinic, a reference center affiliated with Shiraz University of Medical Sciences, Shiraz, Iran to be screened for cervicitis, intraepithelial neoplasia or CC from February 2018 to November 2021 were considered for inclusion in the study. After being informed of the research goals, the participants voluntarily completed a series of examinations consisting of ThinPrep cytology test and HPV DNA detection and typing assay. All participants were aged between 16 and 75 years old and met the inclusion criterion of having a history of sexual intercourse. Further, (1) pregnant women or those who had terminated their pregnancy within 3 months prior to the study, (2) those with mental disorders, (3) vaginal, cervical or uterine hemorrhage, (4) acute infections of lower genital tract, vulva, vagina or cervix, (5) concurrent sexually transmitted diseases, and (6) full or partial HPV vaccination were excluded from the study. Finally, 648 participants were identified as qualified for the study. Moreover, a total of 96 specimens previously screened for HPV infection, including 48 with HPV16 single infection and 48 with HPV18 single infection, were included in the study for variant analysis. Each group consisted of 12 normal, 12 low-grade squamous intraepithelial lesion (LSIL), 12 high-grade squamous intraepithelial lesion (HSIL), and 12 CC specimens. Premalignant/malignant samples in the mentioned groups were formalin-fixed paraffin-embedded (FFPE) tissue biopsies whereas the normal ones were ThinPrep Pap Test specimens. Hematoxylin–eosin staining was performed for all specimens and the diagnoses were confirmed by experienced pathologist.

### ThinPrep cytology testing

For cytology examination, ThinPrep liquid-based cytology samples were referred by physicians to the laboratory based on standard CC screening methodology. Briefly, a sampling brush was used to collect exfoliated cells at the cervical canal and the external aperture of the cervix. Collected cells were stored in a preservation solution. Using a ThinPrep 2000 system, a thin-layer cell smear was prepared for Pap examination. Grading was carried out based on the Bethesda criteria [34] as follows: (I) no intraepithelial lesion or malignancy (NILM); (II) abnormality of squamous epithelial cell including: a, atypical squamous cells (ASCs), consisting of ASCs of undetermined significance (ASC-US) and ASCs that cannot exclude high-grade squamous intraepithelial lesion (ASC-H); b, low-grade squamous intraepithelial lesion (LSIL); c, high-grade squamous intraepithelial lesion (HSIL); and d, squamous cell carcinoma (SCC); 3) glandular epithelial cell abnormality including: a, atypical glandular cells (AGCs), consisting of AGC not otherwise specified (AGC-NOS) and AGC suspicious for neoplasia (AGC-N); b, cervical adenocarcinoma in situ of the cervical canal (AIS); and c, adenocarcinoma (ADC); and 4) other malignant tumors.

### DNA extraction

ThinPrep media containing suspended cells were mixed by inversion and 500 µl-aliquots were separated for DNA extraction using QIAamp DNA mini kit (Qiagen, Hilden, Germany). In the case of FFPE tissue specimens, four 5-μm-thick slices were cut and collected in autoclaved Eppendorf microtubes for each patient. Only one case was sectioned at a time; the microtome blade was changed and the workplace was cleaned with ethanol thoroughly along with the microtome between every two cases to prevent contamination. Furthermore, paraffin blocks without tissue were cut after every real specimen and served as negative controls of DNA extraction process. DNA was extracted from FFPE tissue specimens using QIAamp DNA FFPE Tissue Kit (Qiagen, Hilden, Germany) according to the manufacturer’s instruction. The concentration of the extracted DNA was measured by a NanoDrop (ND-1000) spectrophotometer (peQLab Biotechnologie, Erlangen, Germany). All DNA specimens were stored at − 70 °C until required.

### HPV detection and typing

For HPV DNA detection and genotyping in ThinPrep cytology specimens, the AMPLIQUALITY HPV-TYPE EXPRESS v3.0 (Code: 03-35A-20 M AB, Analitica, Italy) method was used which is based on the amplification of L1 viral region in a Single-Step PCR followed by Reverse Line Blot assay. This method allows the identification of 40 HPV types: 6, 11, 16, 18, 26, 31, 33, 35, 39, 40, 42, 43, 44, 45, 51, 52, 53, 54, 55, 56, 58, 59, 61, 62, 64, 66, 67, 68 (a e b), 69, 70, 71, 72, 73, 81, 82, 83, 84, 87, 89, and 90 and uses the dUTP/UNG system for the prevention of carry-over contamination. Negative controls (5 μl of sterile water instead of DNA) and positive controls (5 μl of the positive control provided in the kit) were included in each PCR run. All procedures were performed according to the manufacturers' instructions. All tests were interpreted manually by two independent readers. A third reader was used in case of disagreement between the results.

### Determination of HPV16 and 18 lineages and sublineages and phylogenetic analysis

Primer sets HPV16-E6-F/HPV16-E6-R and HPV18-E6-F/HPV18-E6-R were used for the amplification of full-length HPV16/18 E6 gene in the DNA samples extracted from the FFPE tissue specimens that had previously tested positive for single infections with either HPV16 or 18 (Additional file 1: Table S1). The amplification of HPV16/18 E6 gene was performed in a 50-μl reaction mixture containing 500 ng of DNA template, 0.5 µM of each primer, and TEMPase Hot Start DNA Polymerase 2 × Master Mix (Ampliqon, Odense, Denmark). PCR amplification cycles included an initial 15-min denaturation at 95 °C, followed by 40 cycles of 95 °C for 45 s, 55 °C for 1 min, and 72 °C for 1 min, and a final elongation at 72 °C for 5 min. A reaction mixture without template DNA, as a negative control, was included in every PCR run. Plasmids containing HPV16 and HPV18 DNA cloned in pBluescript (Manassas, VA, USA) and pBR322 (Manassas, VA), respectively, which were available from a previous study [35], were used as positive controls.

Since using HPV16 E6 G433 and A532 nucleotide sequences could not distinguish between D1 and D4 sublineages, all HPV16 isolates which belonged to the D1/D4 sublineages were further analyzed by PCR assay using 16LCR-F/16LCR-R primer set for the amplification of HPV16 long control region (LCR) according to the previously published protocol [36] (Additional file 1: Table S1). The single nucleotide polymorphism (SNP) C7781T was considered as a diagnostic criterion for differentiation between D1 and D4 sublineages. The specific detection of D1 sublineage was achieved by observing the following six SNP variations: G145T, T286A, A289G, C335T, T350G, and C7781T.

Following visualization on 1.5% agarose gel, the bands of generated amplicons were excised and purified using GF-1 PCR Clean-Up Kit (Vivantis, Malaysia) and were subsequently subjected to sequencing (Sequetech Corp.,Mountain View, CA, USA) in both directions. The obtained sequences are available at http://www.ncbi.nlm.nih.gov/ with GenBank accession numbers from OP572427 to OP572522. All the sequences were analyzed using BLAST software program (http://www.blast.ncbi.nlm.nih.gov/blast/html) and classified into lineages and sublineages according to the prototype reference sequences given in the Papillomavirus Episteme database (http://pave.niaid.nih.gov). Phylogenetic trees were generated using maximum-likelihood method Mega software version 7 [37], and the sublineages were identified based on 0.5–1.0% differences between isolate genomes. Reference HPV16 E6 sequences that were used to construct the phylogenetic branches were collected from the GenBank sequence database and included K02718 (A1), AF536179 (A2), HQ644236 (A3), AF534061 (A4), AF536180 (B1), HQ644298 (B2), KU053910 (B3), KU053914 (B4), AF472509 (C1), HQ644244 (C2), KU053921 (C3), KU053922 (C4), HQ644257 (D1), AY686579 (D2), AF402678 (D3), and KU053933 (D4). Furthermore, in the case of phylogenetic branches for HPV18 E6 gene, reference sequences obtained from the GenBank database included AY262282.1 (A1), EF202146 (A2), EF202147 (A3), EF202151 (A4), GQ180787 (A5), EF202155 (B1), KC470225 (B2), EF202152 (B3), and KC470229 (C1). The robustness of the phylogenetic trees was assessed using 1000 bootstrap repetitions. Furthermore, nucleotide sequences were translated by ExPASy (http://web.expasy.org/translate/) for the determination of amino acid changes.

### Statistical analysis

Data were analyzed using SPSS version 21.0 (SPSS Institute, Chicago, IL, USA). Chi-square test or two-sided Fisher’s exact test was used to analyze the potential association of HPV PCR results, HPV types, and lineages with age, cervical pre-neoplastic lesions, CC, and other categorical factors, where appropriate. p-values less than 0.05 were considered to be statistically significant.

## Results

### Prevalence and distribution of HPV types

**Fig. 1:**
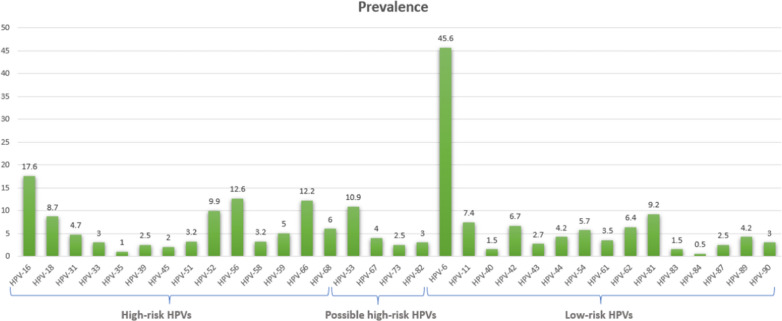
Prevalence of HPV types among HPV-positive individuals

Totally, 648 female participants were included in the study with ages ranging from 16 to 75 years (mean ± SD = 33.83 ± 8.74). Of all the screened subjects, 403 (62.19%) were found to be positive for HPV DNA. HPV typing revealed 33 types among the studied samples. A representative HPV genotyping test strip is provided in Additional file 1: Fig. S1. Those included 14 HR-HPVs (HPV16, 18, 31, 33, 35, 39, 45, 51, 52, 56, 58, 59, 66, 68), 4 pHR-HPVs (HPV53, 67, 73, 82), and 15 LR-HPVs genotypes (HPV6, 11, 40, 42, 43, 44, 54, 61, 62, 81, 83, 84, 87, 89, 90). The distribution of different HPV types among HPV-positive individuals is presented in Fig. 1. The most prevalent HR-HPV type was HPV16 which was detected in 17.6% of HPV-positive cases. Furthermore, HPV6 was found to be the most common LR-HPV type detected in 45.6% of HPV-positive individuals.

**Fig. 2:**
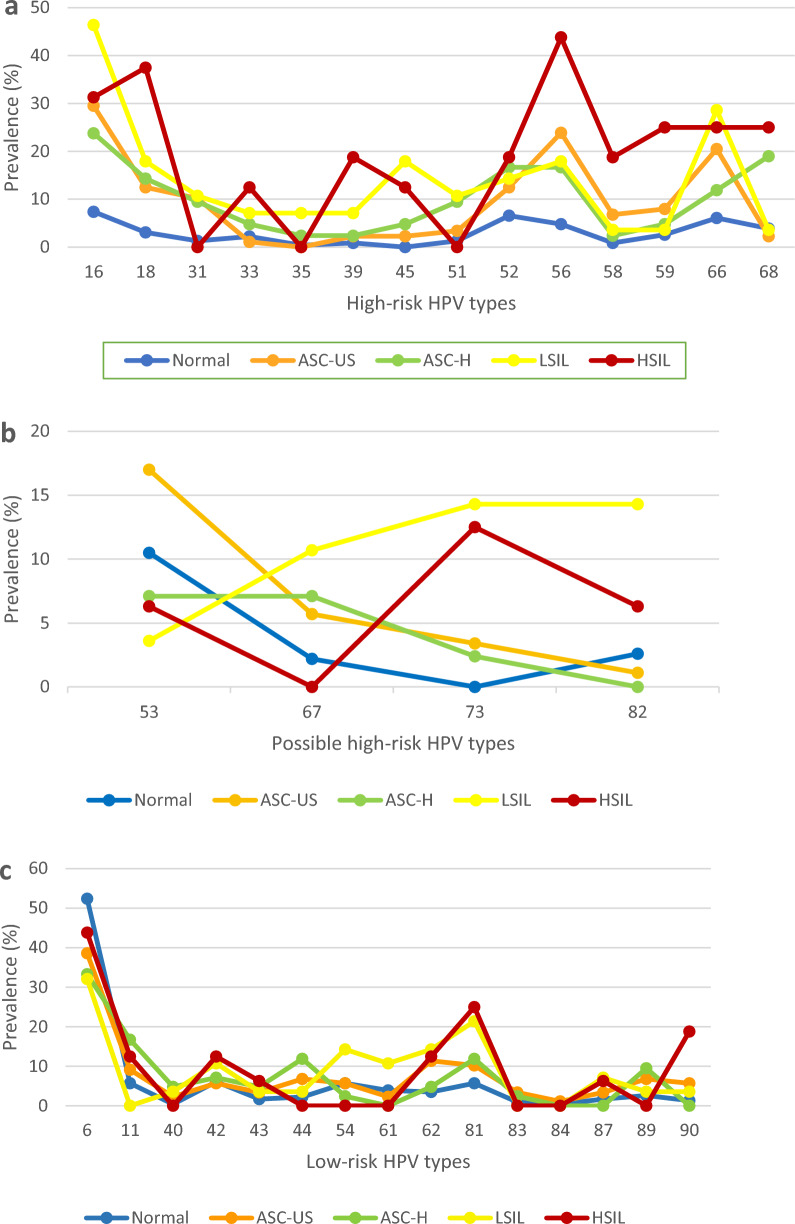
Distribution of high-risk HPV types (**a**), possible high-risk HPV types (**b**), and low-risk HPV types (**c**) in HPV-positive groups of normal, ASC-US, ASC-H, LSIL, and HSIL cervical cytology

The detection rates of HPV testing among individuals with normal, ASC-US, ASC-H, LSIL, and HSIL cervical cytology were 48.9%, 93.6%, 100%, 100% and 100%, respectively (Table 1). The frequency of HPV infection was significantly higher among participants with abnormal cervical cytology than that among individuals with normal cervical cytology (p < 0.0001). The distribution of HR-HPV, pHR-HPV, and LR-HPV types in different cervical cytology groups is presented in Fig. 2.

**Table 1:** Distribution of HPV infection, HPV risk groups, and single/multiple HPV infections among individuals with different cervical cytology grades

	Cervical cytology					P-value
	Normal	ASC-US	ASC-H	LSIL	HSIL	
	N (%)					
**HPV test**						
HPV positive	229 (48.9)	88 (93.6)	42 (100)	28 (100)	16 (100)	P < 0.0001
HPV negative	239 (51.1)	6 (6.4)	0	0	0	
**Risk group**						
High-risk only	35 (15.3)	12 (13.6)	12 (28.6)	8 (28.6)	0	P < 0.0001
Possible high-risk only	12 (5.2)	0	0	0	0	
Low-risk only	135 (59.0)	2 (2.3)	0	0	0	
Multiple risk groups	47 (20.5)	74 (84.1)	30 (75.0)	20 (71.4)	16 (100)	
**Single/multiple infection**						
Single infection	153 (66.8)	8 (9.1)	7 (16.7)	4 (14.3)	0	P < 0.0001
Double infection	51 (22.3)	38 (43.2)	12 (28.6)	5 (17.9)	1 (6.3)	
Triple infection	16 (7.0)	27 (30.7)	19 (45.2)	7 (25.0)	5 (31.3)	
Quadruple infection	3 (1.3)	9 (10.2)	0	5 (17.9)	3 (18.8)	
Quintuple infection	5 (2.2)	0	3 (7.1)	1 (3.6)	4 (25.0)	
Sextuple infection	0	3 (3.4)	0	2 (7.1)	3 (18.8)	
Septuple infection	1 (0.4)	1 (1.1)	0	3 (10.7)	0	
Octuple infection	0	1 (1.1)	1 (2.4)	1 (3.6)	0	
Decuple infection	0	1 (1.1)	0	0	0

Of 403 HPV-positive cases, 172 (42.7%) were found to be infected with a single HPV type while the remaining 231 (57.3%) cases were infected with multiple types of HPV. Multiple HPV infections were significantly more frequent among participants with abnormal cervical cytology than those with normal cervical cytology (p < 0.0001). Moreover, the rate of multiple HPV infections was significantly higher among participants aged above 30 years compared to those with ≤ 30 years of age (p = 0.001). The distribution of single and multiple HPV infections in the groups of different cervical cytology grades and age ranges is presented in Tables 1 and 2, respectively.

**Table 2:** Distribution of different cervical cytology diagnoses, HPV risk groups, and single/multiple HPV infections in different age groups

	Age range						
	≤ 20	21–30	31–40	41–50	51–60	61–70	71–80
	N (%)						
**Cervical cytology**							
Normal	16 (88.9)	207 (83.1)	171 (65.3)	55 (67.1)	19 (61.3)	0	0
ASC-US	1 (5.6)	21 (8.4)	57 (21.8)	12 (14.6)	1 (3.2)	2 (100)	0
ASC-H	1 (5.6)	17 (6.8)	14 (5.3)	8 (9.8)	2 (6.5)	0	0
LSIL	0	4 (1.6)	13 (5.0)	6 (7.3)	5 (16.1)	0	0
HSIL	0	0	7 (2.7)	1 (1.2)	4 (12.9)	0	4 (100)
**Risk group**							
High-risk only	2 (16.7)	34 (20.4)	21 (12.5)	7 (17.9)	3 (23.1)	0	0
Possible high-risk only	0	8 (4.8)	4 (2.4)	0	0	0	0
Low-risk only	2 (16.7)	66 (39.5)	56 (33.3)	13 (33.3)	0	0	0
Multiple risk groups	8 (66.7)	59 (35.3)	87 (51.8)	19 (48.7)	10 (76.9)	0	4 (100)
**Single/multiple infection**							
Single infection	4 (33.3)	89 (53.3)	62 (36.9)	15 (38.5)	2 (15.4)	0	0
Double infection	1 (8.3)	43 (25.7)	48 (28.6)	12 (30.8)	3 (23.1)	0	0
Triple infection	6 (50.0)	16 (9.6)	40 (23.8)	8 (20.5)	3 (23.1)	0	1 (25.0)
Quadruple infection	1 (8.3)	5 (3.0)	10 (6.0)	2 (5.1)	2 (15.4)	0	0
Quintuple infection	0	5 (3.0)	5 (3.0)	0	0	0	3 (75.0)
Sextuple infection	0	3 (1.8)	2 (1.2)	1 (2.6)	2 (15.4)	0	0
Septuple infection	0	3 (1.8)	0	1 (2.6)	1 (7.7)	0	0
Octuple infection	0	2 (1.2)	1 (0.6)	0	0	0	0
Decuple infection	0	1 (0.6)	0	0	0	0	0

Examining the frequency of HPV types in different cytology groups, we found that individuals infected with at least one high-risk or possible high-risk HPV type comprised a significantly larger proportion of abnormal cervical cytology group compared to the normal cervical cytology group (p < 0.0001). Moreover, among the LR-HPV types, HPV6 was found to be the most frequent in all of the cytology groups having infected 36.2%, 33.3%, 32.1%, and 43.8% of individuals in the ASC-US, ASC-H, LSIL, and HSIL group, respectively. Among the HR-HPV types, HPV16 was the most prevalent type in the ASC-US, ASC-H, and LSIL cytology group with the infection rates of 27.7%, 23.8%, and 46.4%, respectively. Interestingly, in the case of the HSIL group, HPV56 was the most frequent HR-HPV type with an infection rate of 43.75%.

With regard to the frequency of HPV types among single and multiple infection cases, HPV6 was the most common LR-HPV type accounting for 47.1% of single infections and present in 44.6% of multiple infection cases. Furthermore, while HPV16, 18, and 52 were the most frequent HR-HPV types responsible for single HPV infections (9.3%, 4.7%, and 4.7% of single infections respectively), HPV16, 56, and 66 were the most frequently detected HR-HPV types among multiple HPV infection cases (present in 23.8%, 20.8%, and 19.5% of multiple infections respectively). HPV53 was found to be the most common pHR-HPV type among both single (6.4%) and multiple infection cases (14.3%).

### Genetic variations of HPV16 and 18 E6 gene regions

**Fig. 3:**
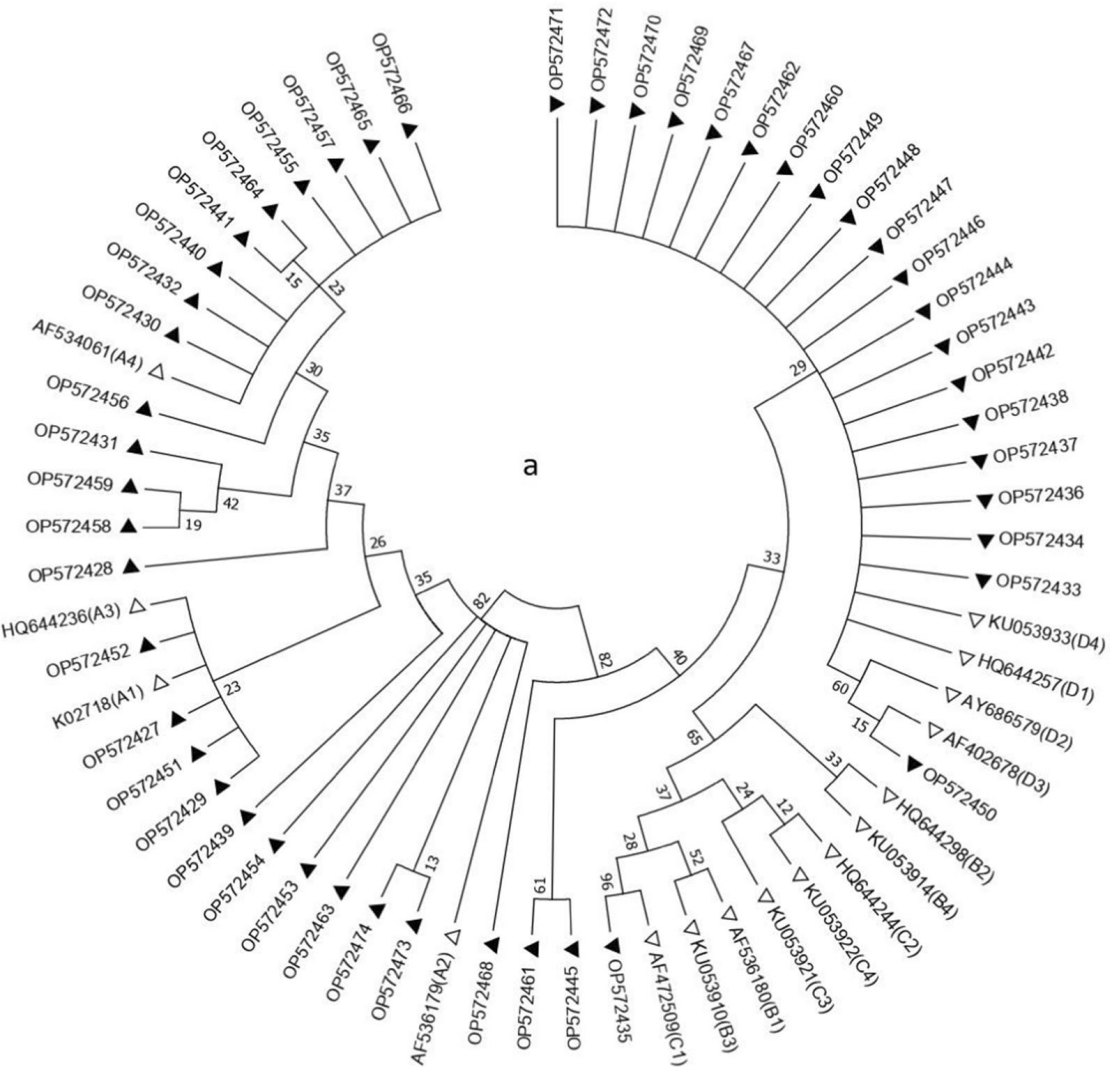

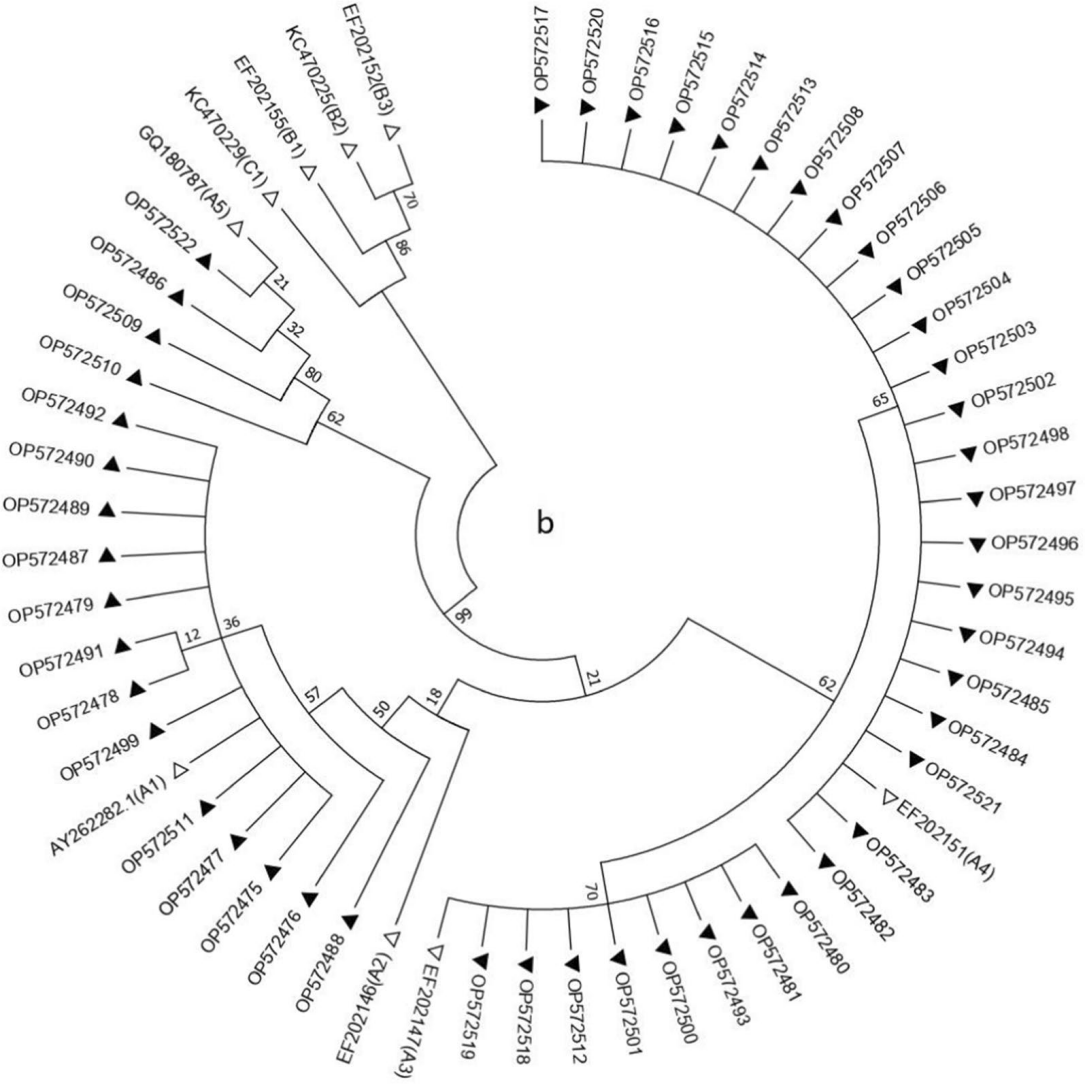
Phylogenetic analysis of HPV16 E6 (**a**) and HPV18 E6 (**b**) gene regions was conducted using the maximum-likelihood method based on the Kimura 2-parameter model with bootstrap resampling (1000 replicates) by the MEGA 6 package [37]. Numbers above the branches indicate the bootstrap values. Ninety-six different nucleotide patterns of studied sequences were indicated by black triangles (GenBank accession numbers OP572427 through OP572474 for HPV16 and OP572475 through OP572522 for HPV18). The accession number of reference sequences of each sublineage used for phylogenetic analysis in this study was indicated by white triangles

Forty-eight HPV16 and 48 HPV18 isolates of the 96 previously screened cases were sequenced across the E6 gene (nt: 83-559 and nt: 105-581) and compared with the corresponding HPV16 and HPV18 E6 reference sequences of each lineage and sublineage (Additional file 1: Tables S2 and S3) Additional file 1: Figs. S2–S7 present DNA Sanger sequencing chromatograms showing sequence polymorphisms throughout the E6 gene region of HPV16 and 18. The sublineage analysis of HPV16 isolates could not distinguish between D1 and D4 based on the HPV16 E6 gene sequences. Therefore, 22 HPV16 isolates were further analyzed by PCR assay for the amplification of HPV16 LCR and subsequent Sanger sequencing of the PCR products. Since C7781T SNP was not detected in any of these isolates, they were all sorted into sublineage D1. Overall, 3 lineages (A, D, and C) and 6 sublineages (A1, A2, A4, C1, D1, and D2) of HPV16, and one lineage and 4 sublineages (A1, A3, A4, and A5) of HPV18 were identified (Tables 3 and 4). The studied HPV16 variants mainly belonged to the D1 sublineage accounting for 45.83% of all HPV16-positive samples, followed by A4 (27.1%), A1 (12.5%), A2 (10.41%), C1 (2.08%), and D2 (2.08%) (Fig. 3a). HPV18 isolates mostly belonged to A4 sublineage comprising 47.91% of all HPV18-positive samples, followed by A1 (27.1%), A3 (16.66%), and A5 (8.33%) (Fig. 3b). The distribution of HPV16 and 18 variants in different cervical cytology grades is presented in Table 5. Neither HPV16 (p = 0.214) nor HPV18 variants (p = 0.579) showed any significant differences in their frequency between normal and premalignant/malignant groups.

**Table 3:** Variations of HPV16 E6 gene from patients with different grades of cervical lesions

No.	Grade of cervical lesion	Type of variant	109	131	132	135	143	145	178	183	286	289	295	335	350	403	442	532	Accession number
		Reference	T	A	G	A	C	G	T	T	T	A	T	C	T	A	A	A	K02718
1	Normal	A1	–	–	–	–	–	–	–	–	–	–	–	–	–	–	–	–	OP572427
2	Normal	A1	–	–	–	–	–	–	–	–	–	–	–	–	–	–	C *	–	OP572428
3	Normal	A1	–	–	–	–	–	–	–	–	–	–	–	–	–	–	–	–	OP572429
4	Normal	A4	–	–	–	–	–	–	G	–	–	–	–	–	–	–	–	–	OP572430
5	Normal	A4	–	–	–	–	–	–	G	–	–	–	–	–	–	–	C *	–	OP572431
6	Normal	A4	–	–	–	–	–	–	G	–	–	–	–	–	–	–	–	–	OP572432
7	Normal	C1	C	–	T	–	G	T	–	–	A	G	–	T	–	G	–	–	OP572433
8	Normal	D1	–	–	–	–	–	T	–	–	A	G	–	T	G	–	–	–	OP572434
9	Normal	D1	–	–	–	–	–	T	–	–	A	G	–	T	G	–	–	–	OP572435
10	Normal	D1	–	–	–	–	–	T	–	–	A	G	–	T	G	–	–	–	OP572436
11	Normal	D1	–	–	–	–	–	T	–	–	A	G	–	T	G	–	–	–	OP572437
12	Normal	D1	–	–	–	–	–	T	–	–	A	G	–	T	G	–	–	–	OP572438
13	LSIL/CIN1	A1	–	G *	–	–	–	–	–	–	–	–	–	–	–	–	–	–	OP572439
14	LSIL/CIN1	A4	–	–	–	–	–	–	G	–	–	–	–	–	–	–	–	–	OP572440
15	LSIL/CIN1	A4	–	–	–	C *	–	–	G	–	–	–	–	–	–	–	–	–	OP572441
16	LSIL/CIN1	D1	–	–	–	–	–	T	–	–	A	G	–	T	G	–	–	–	OP572442
17	LSIL/CIN1	D1	–	–	–	–	–	T	–	–	A	G	–	T	G	–	–	–	OP572443
18	LSIL/CIN1	D1	–	–	–	–	–	T	–	–	A	G	–	T	G	–	–	–	OP572444
19	LSIL/CIN1	D1	–	–	–	–	–	T	–	–	– *	G	–	T	G	–	–	–	OP572445
20	LSIL/CIN1	D1	–	–	–	–	–	T	–	–	A	G	–	T	G	–	–	–	OP572446
21	LSIL/CIN1	D1	–	–	–	–	–	T	–	–	A	G	–	T	G	–	–	–	OP572447
22	LSIL/CIN1	D1	–	–	–	–	–	T	–	–	A	G	–	T	G	–	–	–	OP572448
23	LSIL/CIN1	D1	–	–	–	–	–	T	–	–	A	G	–	T	G	–	–	–	OP572449
24	LSIL/CIN1	D2	–	–	–	–	–	T	–	–	A	G	–	T	G	–	–	G	OP572450
25	HSIL/CIN2-3	A1	–	–	–	–	–	–	–	–	–	–	–	–	–	–	–	–	OP572451
26	HSIL/CIN2-3	A1	–	–	–	–	–	–	A*	–	–	–	–	–	–	–	–	–	OP572452
27	HSIL/CIN2-3	A2	–	G	–	–	–	–	–	–	–	–	–	–	G	–	–	–	OP572453
28	HSIL/CIN2-3	A2	–	G	–	–	–	–	–	–	–	–	G*	–	G	–	–	–	OP572454
29	HSIL/CIN2-3	A4	–	–	–	–	–	–	G	–	–	–	–	–	–	–	–	–	OP572455
30	HSIL/CIN2-3	A4	–	C*	–	–	–	–	G	–	–	–	–	–	–	–	–	–	OP572456
31	HSIL/CIN2-3	A4	–	–	–	–	–	–	G	–	–	–	–	–	–	–	–	–	OP572457
32	HSIL/CIN2-3	A4	–	–	–	–	–	–	G	–	–	–	–	–	–	–	C*	–	OP572458
33	HSIL/CIN2-3	A4	–	–	–	–	–	–	G	–	–	–	–	–	–	–	–	–	OP572459
34	HSIL/CIN2-3	D1	–	–	–	–	–	T	–	–	A	G	–	T	G	–	–	–	OP572460
35	HSIL/CIN2-3	D1	–	–	–	–	–	T	–	–	–*	G	–	T	G	–	–	–	OP572461
36	HSIL/CIN2-3	D1	–	–	–	–	–	T	–	–	A	G	–	T	G	–	–	–	OP572462
37	SCC	A2	–	G	–	–	–	–	–	–	–	–	–	–	G	–	–	–	OP572463
38	SCC	A4	–	–	–	–	–	–	G	G*	–	–	–	–	–	–	–	–	OP572464
39	SCC	A4	–	–	–	–	–	–	G	–	–	–	–	–	–	–	–	–	OP572465
40	SCC	A4	–	–	–	–	–	–	G	–	–	–	–	–	–	–	–	–	OP572466
41	SCC	D1	–	–	–	–	–	T	–	–	A	G	–	T	G	–	–	–	OP572467
42	SCC	D1	–	–	–	–	–	T	–	–	A	G	–	–*	G	–	–	–	OP572468
43	SCC	D1	–	–	–	–	–	T	–	–	A	G	–	T	G	–	–	–	OP572469
44	SCC	D1	–	–	–	–	–	T	–	–	A	G	–	T	G	–	–	–	OP572470
45	SCC	D1	–	–	–	–	–	T	–	–	A	G	–	T	G	–	–	–	OP572471
46	SCC	D1	–	–	–	–	–	T	–	–	A	G	–	T	G	–	–	–	OP572472
47	ADC	A2	–	G	–	–	–	–	–	–	–	–	–	–	G	–	–	–	OP572473
48	ADC	A2	–	G	–	–	–	–	–	–	–	–	–	–	G	–	–	–	OP572474
**Amino acid substitution**			–	R10G	R10G/I	K11T	Q14D	Q14H/D	D25E	I27R	–	–	D64E	H78Y	L83V	–	E113D	–	

The numbers refer to the positions of the nucleotides according to the reference sequence (GenBank accession number K02718). Nucleotide positions in E6 are presented at the top of the table according to the reference sequence. Nucleotide changes are shown by the corresponding letters. Dashes indicate positions at which no variation was found. Amino acid sequence variations are shown at the bottom; amino acid changes whose codons contain more than one nucleotide replacement are marked with /. Asterisks represent the new SNPs detected in the studied HPV16 isolates

*ADC* adenocarcinoma/adenosquamous carcinoma, *CIN* cervical intraepithelial neoplasia, *LSIL* low-grade squamous intraepithelial lesion, *HSIL* high-grade squamous intraepithelial lesion, *SCC* squamous cell carcinoma, *HPV* human papillomavirus

**Table 4:** Variations of HPV18 E6 gene from patients with different grades of cervical lesions

No.	Grade of cervical lesion	Type of variant	104	149	153	232	287	317	377	382	485	549	554	Accession number
		Reference	T	T	C	A	G	T	A	T	T	C	C	AY262282
1	Normal	A1	–	–	–	–	–	–	–	–	–	–	–	OP572475
2	Normal	A1	–	–	–	–	–	G*	–	–	–	–	–	OP572476
3	Normal	A1	–	–	–	–	–	–	–	–	–	–	–	OP572477
4	Normal	A1	–	–	–	–	–	–	–	G*	–	–	–	OP572478
5	Normal	A1	–	–	–	–	–	–	–	–	–	–	–	OP572479
6	Normal	A3	C	–	–	G	–	–	–	–	C	A	–	OP572480
7	Normal	A3	C	–	–	G	–	–	–	–	C	A	–	OP572481
8	Normal	A4	C	–	–	–	–	–	–	–	C	A	–	OP572482
9	Normal	A4	C	–	–	–	–	–	–	–	C	A	–	OP572483
10	Normal	A4	C	–	–	–	C*	–	–	–	C	A	–	OP572484
11	Normal	A4	C	–	–	–	–	–	–	–	C	A	–	OP572485
12	Normal	A5	C	C	–	–	–	–	G	–	C	A	–	OP572486
13	LSIL/CIN1	A1	–	–	–	–	–	–	–	–	–	–	–	OP572487
14	LSIL/CIN1	A1	–	C*	–	–	–	–	–	–	–	–	–	OP572488
15	LSIL/CIN1	A1	–	–	–	–	–	–	–	–	–	–	–	OP572489
16	LSIL/CIN1	A1	–	–	–	–	–	–	–	–	–	–	–	OP572490
17	LSIL/CIN1	A1	–	–	–	–	–	–	–	–	–	–	T*	OP572491
18	LSIL/CIN1	A1	–	–	–	–	–	–	–	–	–	–	–	OP572492
19	LSIL/CIN1	A3	C	–	–	G	–	–	–	–	C	A	–	OP572493
20	LSIL/CIN1	A4	C	–	–	–	–	–	–	–	C	A	–	OP572494
21	LSIL/CIN1	A4	C	–	–	–	–	–	–	–	C	A	–	OP572495
22	LSIL/CIN1	A4	C	–	–	–	–	–	–	–	C	A	–	OP572496
23	LSIL/CIN1	A4	C	–	–	–	–	–	–	–	C	A	–	OP572497
24	LSIL/CIN1	A4	C	–	–	–	–	–	–	–	C	A	–	OP572498
25	HSIL/CIN2-3	A1	–	–	–	–	–	–	–	–	–	–	–	OP572499
26	HSIL/CIN2-3	A3	C	–	–	G	–	–	–	–	C	A	–	OP572500
27	HSIL/CIN2-3	A3	C	–	–	G	–	–	–	–	C	A	–	OP572501
28	HSIL/CIN2-3	A4	C	–	–	–	–	–	–	–	C	A	–	OP572502
29	HSIL/CIN2-3	A4	C	–	–	–	–	–	–	–	C	A	–	OP572503
30	HSIL/CIN2-3	A4	C	–	–	–	–	–	–	–	C	A	–	OP572504
31	HSIL/CIN2-3	A4	C	–	–	–	–	–	–	–	C	A	–	OP572505
32	HSIL/CIN2-3	A4	C	–	–	–	–	–	–	–	C	A	–	OP572506
33	HSIL/CIN2-3	A4	C	–	–	–	–	–	–	–	C	A	–	OP572507
34	HSIL/CIN2-3	A4	C	–	–	–	–	–	–	–	C	A	–	OP572508
35	HSIL/CIN2-3	A5	C	C	T*	–	–	–	G	–	C	A	–	OP572509
36	HSIL/CIN2-3	A5	C	C	–	–	–	–	G	–	–*	–*	–	OP572510
37	SCC	A1	–	–	–	–	–	–	–	–	–	–	–	OP572511
38	SCC	A3	C	–	–	G	–	–	–	–	C	A	–	OP572512
39	SCC	A4	C	–	–	–	–	–	–	–	C	A	–	OP572513
40	SCC	A4	C	–	–	–	–	–	–	–	C	A	–	OP572514
41	SCC	A4	C	–	–	–	–	–	–	–	C	A	–	OP572515
42	SCC	A4	C	–	–	–	–	–	–	–	C	A	–	OP572516
43	SCC	A4	C	–	–	–	–	–	–	–	C	A	–	OP572517
44	ADC	A3	C	–	–	G	–	–	–	–	C	A	–	OP572518
45	ADC	A3	C	–	–	G	–	–	–	–	C	A	–	OP572519
46	ADC	A4	C	–	–	–	–	–	–	–	C	A	–	OP572520
47	ADC	A4	C	–	–	–	–	–	–	–	C	A	–	OP572521
48	ADC	A5	C	C	–	–	–	–	G	–	C	A	–	OP572522
**Amino acid substitution**			-	-	–	E33G	–	F71L	–	L93R	–	–	–	

The numbers refer to the positions of the nucleotides according to the reference sequence (GenBank accession number AY262282). Nucleotide positions in E6 are presented at the top of the table according to the reference sequence. Nucleotide changes are shown by the corresponding letters. Dashes indicate positions at which no variation was found. Amino acid sequence variations are shown at the bottom; amino acid changes whose codons contain more than one nucleotide replacement are marked with /. Asterisks represent the new SNPs detected in the studied HPV18 isolates

*ADC* adenocarcinoma/adenosquamous carcinoma, *CIN* cervical intraepithelial neoplasia, *LSIL* low-grade squamous intraepithelial lesion, *HSIL* high-grade squamous intraepithelial lesion, *SCC* squamous cell carcinoma, *HPV* human papillomavirus

**Table 5:** Distribution of HPV16 and HPV18 variants in different cervical cytology grades

	Cervical cytology			
	Normal	LSIL	HSIL	SCC/ADC
	N (%)			
HPV16 lineage				
A1	3 (25)	1 (8.3)	2 (16.7)	0
A2	0	0	2 (16.7)	3 (25)
A4	3 (25)	2 (16.7)	5 (41.7)	3 (25)
C1	1 (8.3)	0	0	0
D1	5 (41.7)	8 (66.7)	3 (25)	6 (50)
D2	0	1 (8.3)	0	0
HPV18 lineage				
A1	5 (41.7)	6 (50)	1 (8.3)	1 (8.3)
A3	2 (16.7)	1 (8.3)	2 (16.7)	3 (25)
A4	4 (33.3)	5 (41.70	7 (58.3)	7 (58.3)
A5	1 (8.3)	0	2 (16.7)	1 (8.3)

Nucleotide variations in the E6 gene were observed in 12 HPV16 isolates (25%) when compared to the reference sequences of each sublineage. New SNPs detected in the studied HPV16 isolates from Iranian patients included A131G (A1), A131C (A4), A135C (A4), T178A (A1), T183G (A4), T286T (D1), T295G (A2), and A442C (A1 and A4). The remaining substitutions at the positions of A286T (D1) and T335C (D1) detected in three isolates were found to be silent mutations (Table 3). In addition, amino acid changes in the E6 gene had occurred in 45 isolates (93.75%). L83V was the most common amino acid change (58.33%), followed by Q14H/D (50%) and H78Y (47.91%) (Table 3). Among HPV18 isolates, eight new SNPs were detected throughout the E6 gene including T149C (A1), C153T (A5), G287C (A4), T485T (A5), C549C (A5), and C554T (A1), all of which were silent mutations except for T317G (A1) and T382G (A1) which were found to be missense mutations. Furthermore, amino acid changes in the E6 gene had occurred in 10 HPV18 isolates (20.83%) among which, the most common amino acid change was E33G (16.66%), followed by F71L (2.1%) and L93R (2.1%) (Table 4). No deletions or insertions were found in any of the HPV16 or 18 E6 genes.

## Discussion

This is the first investigation of the presence of HPV types in a large-scale screen of liquid-based cytology samples from Iranian population in the southwest. The study population was comprised of gynecological outpatients including symptomatic and asymptomatic women. The vast majority of Iranian published data include FFPE tissue specimens in hospitals and research centers. However, the current study focused on ThinPrep cytology samples from patients in Shiraz, the capital city of Fars Province. In this study, the overall HPV-positive rate was 62.19%, a rate similar to but slightly higher than that in a recent cross-sectional retrospective study (52.25%) by Rezaee Azhar et al. on female outpatients referred to the medical laboratories of Tehran Metropolitan, Iran [33]. Previously, in the largest Iranian study including 10,266 samples collected from 31 Iranian provinces, Mobini Kesheh et al. found 45.9% (n = 8351) of women positive for HPV DNA [20]. Reports from different parts of the world indicate an overall HPV prevalence ranging from 9.9 to 49.1% [38, 39]. Furthermore, consistent with previously published reports from Iran, the prevalence of HPV has been reported to range from 5.5 to 9.4% in normal cytology specimens, 61.7 to 65.3% in CIN I–III specimens, and 75.2 to 87% in CC specimens with various study populations and methodologies [25–32]. In our study, HPV prevalence in normal, ASC-US, ASC-H, LSIL, and HSIL cervical cytology samples were 48.9%, 93.6%, 100%, 100%, and 100%, respectively which appeared to be higher than not only the overall prevalence previously reported in Iran, but also previous reports from regional countries and most of other countries worldwide [40–43]. On the other hand, the prevalence of HPV infection in the present study is consistent with a study by Schmit et al. [8] reporting the presence of any of 51 investigated genital HPV types in 33.3%, 83.1%, 98.2%, and 100% of normal, ASC-US, LSIL, and HSIL cervical cytology samples, respectively. Taken together, the present study obviously shows that the prevalence of HPV infection in the southwest of Iran is high. This may be due to the fact that this study was cross-sectional and since HPV infections can be transient and cleared up by the immune system, the prevalence of HPV may accordingly change over time. Furthermore, there is evidence that the distribution of HPV types varies by region and ethnicity [44, 45] which might be another explanation for the current finding. In addition, many of the differences observed in the prevalence of HPV infections among different studies can be attributable to the methodological differences in the PCR-based assays used including the size of the PCR product, primer sets, reaction conditions, the efficiency of the polymerase enzyme, the potential of the HPV DNA spectrum amplified to detect multiple types, and even the type (frozen/FFPE/cytology specimens) and quality of the clinical specimens, causing variations in the specificity and sensitivity of the assays [46]. Herein, we have used methodological assays that are among the few ones capable of detecting 40 HPV types including HR-HPVs, pHR-HPVs, and LR-HPVs, and are also more sensitive than those used in previous studies in the region, allowing the identification of HPV types in specimens with low viral loads. HPV infection is responsible for almost 100% of cervical SCCs. The underestimation of HPV prevalence in most reports is attributable to the technical limitations of the corresponding studies [47]. However, the possibility of bias in the estimation of HPV prevalence should be taken into account since to date, Iran has not had an organized national and regular CC screening program and the cervical samples evaluated in this study were collected only from the referred volunteer outpatients attending routine gynecological visits for cytology-based screening which could not have reflected the real virus epidemiology among the general population.

In the present study, the five most prevalent HR-HPV types in ThinPrep cytology samples were HPV16 (17.6%), 56 (12.6%), 66 (12%), 52 (9.9%), and 18 (8.7%) which comprised 61% of the total HR-HPV-positive samples. In addition, HPV6 (45.6%), 81 (9.2%), and 11 (7.4%) were found to be the most dominant LR-HPV types. In the largest study ever conducted in Iran, the most common HPV types reported by Mobini Kesheh et al. were HPV6 (43.3%), HPV16 (16.6%), HPV11 (11.4%), and HPV52 (9.6%) [20]. Another study reported the five most common types to be HPV6, HPV11, HPV16, HPV51, and HPV53 [48]. In a study by Bitarafan et al. on 12,076 Iranian women, the five most common HR-HPV types were as follows: HPV16 (16.98%), HPV52 (8.8%), HPV18 (7.69%), HPV39 (7.63%), and HPV31 (7.45%) [19]. The most similar results to our study reported from Iran were those by Rezaee Azhar et al. revealing HPV16 (12%), 66 (7%), 18 (6%), 31 (5%), and 52 (5%) as the five most prevalent HR-HPV types and HPV6 (32%) and 11 (6%) as the most prevalent LR-HPV types [33]. Interestingly, we found HPV81, a rarely reported type from Iran, to be the second most prevalent LR-HPV type and detected HPV90 and 87, first reported types in Iran, in women with both normal and abnormal cytology; a finding that is consistent with two studies from Qatar, one of the nearest neighboring countries to Fars province in Iran, which reported HPV81, 90, and 11 as the most prevalent LR-HPV types among Arab women [40, 49]. The current finding is another piece of evidence indicating that the geographic distributions of HPV types vary greatly. Given that current HPV vaccines only confer protection against certain HPV types, regional variations in the distribution of HPV types can alter the effectiveness of vaccination. Accordingly, the investigation of HPV distribution patterns can be critical to the development and application of HPV vaccines. In Iran, the 2-valent and 4-valent vaccines are not currently included in the national vaccination program. Furthermore, although the 9-valent vaccine has been approved by the Ministry of Food and Drug Safety and is available as a non-national immunization program vaccine, it does not provide immunity against the HR-HPV types 35, 39, 51, 56, 59, 66, and 68, some of which are highly prevalent in our population. On the other hand, given the implication of HPV in multiple types of cancer aside from CC, including head and neck carcinomas and esophageal carcinoma which is among the most common malignancies in the Iranian population [50, 51], the development of novel vaccines with the potential for immunization against major circulating HR-HPV types in the region including 56, 59, 66, and 68 deserves special consideration.

Unlike single infections, multiple HPV infections have been reported to be associated with increased risk of high-grade lesions and cancer [52]. Therefore, investigating the prevalence and patterns of multiple HPV infections can provide a better understanding of their role in carcinogenesis and the prognosis of patients with persistent infection. A study by Lee et al. has reported an association between multiple HPV infections and an increased risk of CC [53]. Further, Schmitt et al. have reported that HPV co-infection lengthens the course of infection [54]. In line with previously reported studies [41, 55], patients with cytological findings of ASC-US, ASC-H, LSIL, and HSIL showed higher multiple HR-HPV-positive rates (89.1%) than women with normal cytological results (66.8%). Cytological findings have shown high rates of multiple HR-HPV infections in low-grade as well as high-grade lesions, suggesting that multiple HR-HPV infections are associated with all stages of cervical lesions. These results are consistent with those of other cross-sectional and prospective studies [55–57].

Data on HPV variants are of value in HPV diagnosis, developing vaccines, and therapeutic approaches to control virus-induced pathological damage. The oncogenicity of different HPV variants may vary among different populations with distinct distribution of human leukocyte antigens (HLA) alleles [58, 59]. To our knowledge, this is the first study characterizing genetic variations in the E6 gene region of HPV16 and 18 variants simultaneously, in normal, LSIL, HSIL, and CC specimens from women living in the southwest of Iran. Given the critical role of E6 gene in cell immortalization and malignancy, it was selected for the classification of the intra-typic HPV16 and HPV18 variants. In the current work, D1 followed by A4 sublineages were found to be the major HPV16 variants which is in line with previous studies from other parts of Iran [21, 24]. Moreover, we were able to identify A1, A2, and C1 sublineages which were not previously detected simultaneously in either of the two previous studies from Iran. We also identified 10 new nucleotide substitutions in the sequence of HPV16 E6 gene which were not previously reported in the country and accordingly submitted to GenBank. When aligned with E6 gene sequences available in the database, we noticed that all of the detected substitutions were previously reported by other investigators especially from Asia.

There is strong evidence that HPV16 lineage D is associated with CIN3 + with threefold higher risk than lineage A. It is also associated with a higher risk of persistent infection, invasive and glandular high-grade lesions, and CC development than lineage A [60, 61]. In addition, HPV16 lineage D infection results in higher rates of genomic integration compared to other HPV16 lineages [62]. Due to the high prevalence of HPV16 lineage D variants in the Iranian papulation, it seems that they are most likely at high risk of cancer development and progression which necessitates an immediate action for HPV vaccination. With regard to the distribution of HPV18 sublineages, A4 was the most frequent sublineage detected in the current study. This finding is consistent with the results of two previous studies which also reported HPV18 A4 sublineage to be the predominant variant in Iran [22, 23]. However, there is a single study from Iran reporting A3 as the most prevalent sublineage in the country [21]. In general, our finding is in line with the global distribution pattern of HPV18 variants with a predominance of the A lineage in most parts of the world except sub-Saharan Africa. More specifically, A3 and A4 sublineages strongly predominate in South/Central Asia, northern Africa, Europe, and South/Central America [17]. While the majority of A5 isolates have been detected in Africa [17], this sublineage was also detected in our study and previously reported by another study in Iran [23] and a study in Saudi Arabia [63] which shares a maritime border with Iran. Previous studies have suggested that the distribution of HPV18 variants is different between ADC and SCC cases [64, 65]. However, due to the small number of HPV18-positive ADC cases included in our study, no significant difference in HPV18 variant distribution was observed between ADC and SCC cases. On the other hand, in a study with a larger sample size including 81 ADC cases, each matched with two SCC cases in terms of country and age, no difference in HPV18 variant distribution, either overall or in any of the regions, was found [17]. In our population, eight substitutions were found in HPV18 variants which were not previously reported in the country. Following further examination, we found these mutations previously reported by Korean researchers [18], a finding that supports the geographical distribution of HPV lineages. Previous studies have found that HPV18 lineage B could be associated with a higher risk of CC than lineage A, and a higher risk of persistence and progression [60, 66]. In contrast, other studies have reported that no significant difference was observed in the risk of pre-invasive lesions among HPV18 lineages (A, B, and C) [17, 67]. Since we did not detect any non-A lineages of HPV18 in our study, it was not feasible to analyze the relationship between HPV18 lineages and the risk of cervical pre-invasive and invasive lesions. However, our study revealed no statistically significant association between HPV18 A lineage and cervical lesions in Iran. This result may have been affected by the small sample size of HPV18-related cervical lesions in our study.

Generally, the present work had a few limitations, the most important of which was recruiting patients from a single center. In order to confirm the results of this study, multicenter studies are required. Further, since the results from cross-sectional studies may be misleading, longitudinal studies with focus on persistent infections with each specific HPV type need to be performed to further investigate the role of each HPV type in CC development. In addition, the considerable difference observed in the frequency of different HPV types among former studies highlights the need for further investigations to provide additional information on the geographical distribution of HPV types and variants in Iran over time using standard methodological techniques for HPV detection and typing. Such data can help to decide upon the best diagnostic and therapeutic approaches, evaluate the efficiency of currently used vaccines, and develop new generations of them.

## Conclusions

To sum up, the present study suggests that the prevalence of HPV infection in women of all age groups with or without premalignant lesions in the southwestern Iran is high. Our data also show that the predominant HPV types in the southwest of Iran may differ from those detected in other parts of the country. Findings from this study illustrate the necessity of initiating HPV vaccination for the general population and developing national cervical screening programs as well as targeted education to the younger population in order to encourage the application of infection control measures. Moreover, the identification of emerging HPV types that are not covered even by the new 9-valent HPV vaccine raises awareness about potentially important HPV variants. Regarding the approach to cervical screening for cancerous and precancerous lesions in the region, it is best to use a combination of thinPrep cytology test and HPV detection assays in order to improve the accuracy of the screening. Finally, accurate data on the geographic distribution of different HPV types and HPV16/18 variants can be beneficial for developing diagnostic probes and targeted HPV vaccines for Iranian populations.

### Supplementary Information

**Additional file 1**: **Table S1.** The sequences of primers used for the amplification of HPV16 and HPV18 E6 gene. **Table S2.** HPV16 sublineages based on distinguishing positions in E6 gene region. **Table 3.** HPV18 sublineages based on distinguishing positions in E6 gene region. **Fig. S1. **A representative reverse line blot HPV genotyping test strip using AMPLIQUALITY HPV-TYPE EXPRESS kit showing colored bands corresponding to HPV6 and 56. The strip is coated with a staining control band, an amplification control of the housekeeping thiosulfate sulfurtransferase (TST) gene band, and a universal HPV band. **Fig. S2. **Direct DNA Sanger sequencing chromatogram showing a sequence polymorphism (highlighted in blue) in E6 gene region of HPV16 lineage A1 isolate HAM14 (GenBank accession Number: OP572428). **Fig. S3. **Direct DNA Sanger sequencing chromatogram showing a sequence polymorphism (highlighted in blue) in E6 gene region of HPV16 lineage A4 isolate HAM27 (GenBank accession Number: OP572441). **Fig. S4. **Direct DNA Sanger sequencing chromatogram showing a sequence polymorphism (highlighted in blue) in E6 gene region of HPV16 lineage A2 isolate HAM40 (GenBank accession Number: OP572454). **Fig. S5. **Direct DNA Sanger sequencing chromatogram showing a sequence polymorphism (highlighted in blue) in E6 gene region of HPV18 lineage A1 isolate HAM62 (GenBank accession Number: OP572476). **Fig. S6. **Direct DNA Sanger sequencing chromatogram showing a sequence polymorphism (highlighted in blue) in E6 gene region of HPV18 lineage A1 isolate HAM77 (GenBank accession Number: OP572491) iosulfate sulfurtransferase (TST) gene band, and a universal HPV band.

## Data Availability

All data generated or analysed during this study are included in this published article and its supplementary information files.

## Abbreviations

ADC: Adenocarcinoma
ASC: Atypical squamous cell
ASC-H: Atypical squamous cells that cannot exclude high-grade squamous intraepithelial lesion
ASC-US: Atypical squamous cells of undetermined significance
CC: Cervical cancer
CIN: Cervical intraepithelial neoplasia
FFPE: Formalin-fixed paraffin-embedded
HSIL: High-grade squamous intraepithelial lesion
HPV: Human papillomavirus
HR-HPV: High-risk HPV
LR-HPV: Low-risk HPV
LSIL: Low-grade squamous intraepithelial lesion
pHR-HPV: Possible high-risk
SCC: Squamous cell carcinoma
