# Supplementary material for: Type distribution of human papillomaviruses in ThinPrep cytology samples and HPV16/18 E6 gene variations in FFPE cervical cancer specimens in Fars province, Iran
Source: Cancer Cell Int. 2023 Aug 11;23:166. doi: 10.1186/s12935-023-03011-8 (PMC10422805; doi:10.1186/s12935-023-03011-8)
Supplement: Supplementary file 1 — Additional file 1: Table S1. The sequences of primers used for the amplification of HPV16 and HPV18 E6 gene. Table S2. HPV16 sublineages based on distinguishing positions in E6 gene region. Table 3. HPV18 sublineages based on distinguishing positions in E6 gene region. Fig. S1. A representative reverse line blot HPV genotyping test strip using AMPLIQUALITY HPV-TYPE EXPRESS kit showing colored bands corresponding to HPV6 and 56. The strip is coated with a staining control band, an amplification control of the housekeeping thiosulfate sulfurtransferase (TST) gene band, and a universal HPV band. Fig. S2. Direct DNA Sanger sequencing chromatogram showing a sequence polymorphism (highlighted in blue) in E6 gene region of HPV16 lineage A1 isolate HAM14 (GenBank accession Number: OP572428). Fig. S3. Direct DNA Sanger sequencing chromatogram showing a sequence polymorphism (highlighted in blue) in E6 gene region of HPV16 lineage A4 isolate HAM27 (GenBank accession Number: OP572441). Fig. S4. Direct DNA Sanger sequencing chromatogram showing a sequence polymorphism (highlighted in blue) in E6 gene region of HPV16 lineage A2 isolate HAM40 (GenBank accession Number: OP572454). Fig. S5. Direct DNA Sanger sequencing chromatogram showing a sequence polymorphism (highlighted in blue) in E6 gene region of HPV18 lineage A1 isolate HAM62 (GenBank accession Number: OP572476). Fig. S6. Direct DNA Sanger sequencing chromatogram showing a sequence polymorphism (highlighted in blue) in E6 gene region of HPV18 lineage A1 isolate HAM77 (GenBank accession Number: OP572491) iosulfate sulfurtransferase (TST) gene band, and a universal HPV band. [file 12935_2023_3011_MOESM1_ESM.docx]

**Additional file 1**

**Table S1.** The sequences of primers used for the amplification of HPV16 and HPV18 E6 gene

| Reference | Product size (bp) | Nucleotide Positions * | Sequences (5’ to 3’) | Primer Name |
| --- | --- | --- | --- | --- |
| (24) | 584 | 57-78 | CCGGTTAGTATAAAAGCAGACA | HPV16-E6-F |
|  |  | 640-619 | GCTCATAACAGTAGAGATCAGT | HPV16-E6-R |
| (22) | 667 | 80-100 | GATGTGAGAAACRCACCACAA | HPV18-E6-F |
|  |  | 746-726 | GTCGGGCTGGTAAATGTTGAT | HPV18-E6-R |
| (26) | 483 | 7525–7544 | ACTTGTACGTTTCCTGCTTG | HPV16-LCR-F |
|  |  | 85–103 | TGCAGTTCTCTTTTGGTGC | HPV16-LCR-R |

R=A or G; *the numbers refer to the position of the nucleotides according to the reference sequence (GenBank accession number K02718 for HPV16 and GenBank accession number AY262282.1 for HPV18).

**Table S2.** HPV16 sublineages based on distinguishing positions in E6 gene region

| Sublineage | Nucleotide position | | | | | | | | | | | | | | | | | | | Accession Number |
| --- | --- | --- | --- | --- | --- | --- | --- | --- | --- | --- | --- | --- | --- | --- | --- | --- | --- | --- | --- | --- |
|  | **83** | **109** | **131** | **132** | **135** | **137** | **143** | **145** | **178** | **276** | **285** | **286** | **289** | **295** | **335** | **350** | **403** | **433** | **532** |  |
| A1 | A | T | A | G | A | T | C | G | T | A | C | T | A | T | C | T | A | G | A | K02718 |
| A2 | - | - | G | - | - | - | - | - | - | - | - | - | - | - | - | G | - | - | - | AF536179 |
| A3 | - | - | - | - | - | - | - | - | - | G | - | - | - | - | - | - | - | - | - | HQ644236 |
| A4 | - | - | - | - | - | - | - | - | G | - | - | - | - | - | - | - | - | - | - | AF534061 |
| B1 | C | - | - | C | - | - | G | T | - | - | - | A | G | - | T | - | - | - | - | AF536180 |
| B2 | - | - | G | - | - | - | G | T | - | - | - | A | G | - | T | - | - | - | - | HQ644298 |
| B3 | C | - | - | - | - | - | G | T | - | - | - | A | G | G | T | - | - | - | - | [KU053910](http://www.ncbi.nlm.nih.gov/nuccore/KU053910?report=genbank) |
| B4 | - | - | G | - | - | - | G | T | - | - | - | A | G | G | T | G | - | - | - | [KU053914](http://www.ncbi.nlm.nih.gov/nuccore/KU053914?report=genbank) |
| C1 | - | C | - | T | - | - | G | T | - | - | - | A | G | - | T | - | G | - | - | AF472509 |
| C2 | - | - | - | - | - | A | G | T | - | - | G | A | G | - | T | - | - | - | - | [HQ644244](http://www.ncbi.nlm.nih.gov/nuccore/HQ644244?report=genbank) |
| C3 | - | - | - | - | - | - | G | T | - | - | - | A | G | - | T | - | - | - | - | [KU053921](http://www.ncbi.nlm.nih.gov/nuccore/KU053921?report=genbank) |
| C4 | - | - | - | - | - | - | G | T | - | - | - | A | G | - | T | - | - | - | - | [KU053922](http://www.ncbi.nlm.nih.gov/nuccore/KU053922?report=genbank) |
| D1 | - | - | - | - | - | - | - | T | - | - | - | A | G | - | T | G | - | - | - | HQ644257 |
| D2 | - | - | - | - | - | - | - | T | - | - | - | A | G | - | T | G | - | - | G | AY686579 |
| D3 | - | - | - | - | - | - | - | T | - | - | - | A | G | - | T | G | - | A | G | AF402678 |
| D4 | - | - | - | - | - | - | - | T | - | - | - | A | G | - | T | G | - | - | - | [KU053933](http://www.ncbi.nlm.nih.gov/nuccore/KU053933?report=genbank) |

Nucleotide sequence variations of E6 gene region among HPV16 sublineages according to the reference sequences of each variant accessioned by NCBI nucleotide database.

**Table S3.** HPV18 sublineages based on distinguishing positions in E6 gene region

| Sublineage | Nucleotide position | | | | | | | | | | | | | | | | | | | | Accession Number |
| --- | --- | --- | --- | --- | --- | --- | --- | --- | --- | --- | --- | --- | --- | --- | --- | --- | --- | --- | --- | --- | --- |
|  | **104** | **149** | **153** | **232** | **251** | **266** | **287** | **317** | **342** | **374** | **377** | **382** | **437** | **485** | **491** | **548** | **549** | **551** | **554** | **593** |  |
| A1 | T | T | C | A | T | G | G | T | C | G | A | T | G | T | C | A | C | A | C | C | AY262282.1 |
| A2 | - | - | - | - | - | - | - | - | - | - | - | - | - | C | - | - | A | - | - | - | [EF202146](http://www.ncbi.nlm.nih.gov/nuccore/EF202146?report=genbank) |
| A3 | C | - | - | G | - | - | - | - | - | - | - | - | - | C | - | - | A | - | - | - | [EF202147](http://www.ncbi.nlm.nih.gov/nuccore/EF202147?report=genbank) |
| A4 | C | - | - | - | - | - | - | - | - | - | - | - | - | C | - | - | A | - | - | - | [EF202151](http://www.ncbi.nlm.nih.gov/nuccore/EF202151?report=genbank) |
| A5 | C | C | - | - | - | - | - | - | - | - | G | - | - | C | - | - | A | - | - | - | [GQ180787](http://www.ncbi.nlm.nih.gov/nuccore/GQ180787?report=genbank) |
| B1 | - | - | - | - | C | A | - | C | T | A | - | - | - | C | A | G | A | G | - | T | [EF202155](http://www.ncbi.nlm.nih.gov/nuccore/EF202155?report=genbank) |
| B2 | - | - | - | - | C | A | - | - | - | A | - | - | - | C | A | G | A | - | - | T | [KC470225](http://www.ncbi.nlm.nih.gov/nuccore/KC470225?report=genbank) |
| B3 | - | - | - | - | C | A | - | - | - | A | - | - | - | C | A | G | A | - | - | T | [EF202152](http://www.ncbi.nlm.nih.gov/nuccore/EF202152?report=genbank) |
| C1 | - | - | - | - | C | A | - | - | - | A | - | - | A | C | - | G | A | - | - | T | [KC470229](http://www.ncbi.nlm.nih.gov/nuccore/KC470229?report=genbank) |

Nucleotide sequence variations of E6 gene region among HPV18 sublineages according to the reference sequences of each variant accessioned by NCBI nucleotide database.


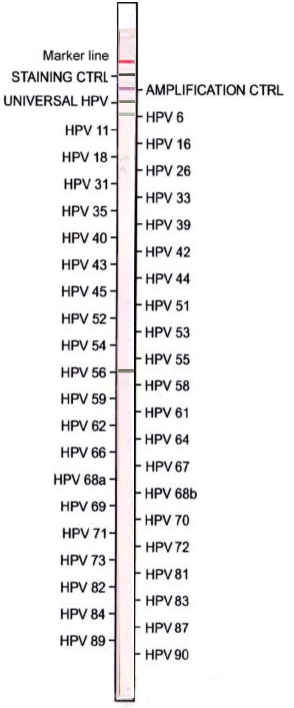


**Fig S1.** A representative reverse line blot HPV genotyping test strip using AMPLIQUALITY HPV-TYPE EXPRESS kit showing colored bands corresponding to HPV6 and 56. The strip is coated with a staining control band, an amplification control of the housekeeping thiosulfate sulfurtransferase (TST) gene band, and a universal HPV band


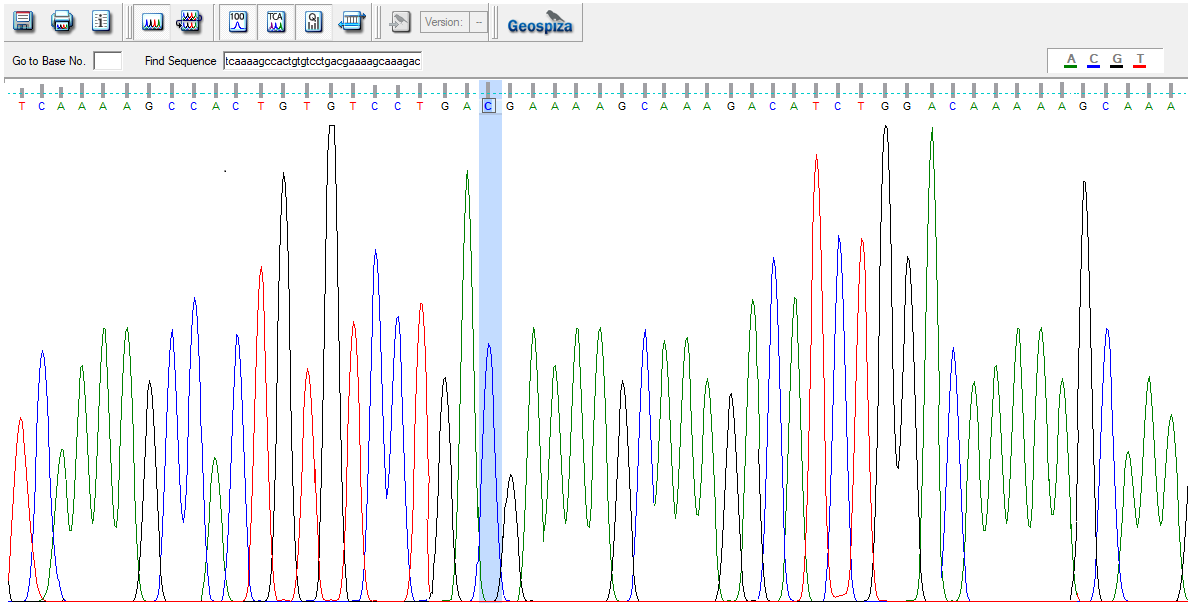


**Fig S2.** Direct DNA Sanger sequencing chromatogram showing a sequence polymorphism (highlighted in blue) in E6 gene region of HPV16 lineage A1 isolate HAM14 (GenBank accession Number: OP572428)


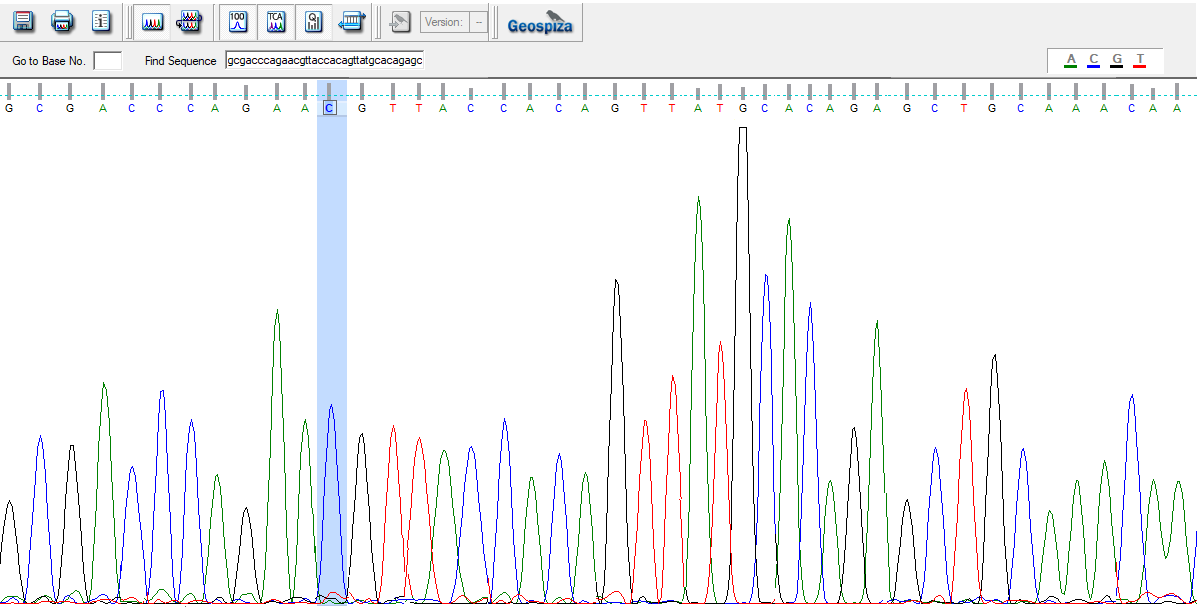


**Fig S3.** Direct DNA Sanger sequencing chromatogram showing a sequence polymorphism (highlighted in blue) in E6 gene region of HPV16 lineage A4 isolate HAM27 (GenBank accession Number: OP572441)


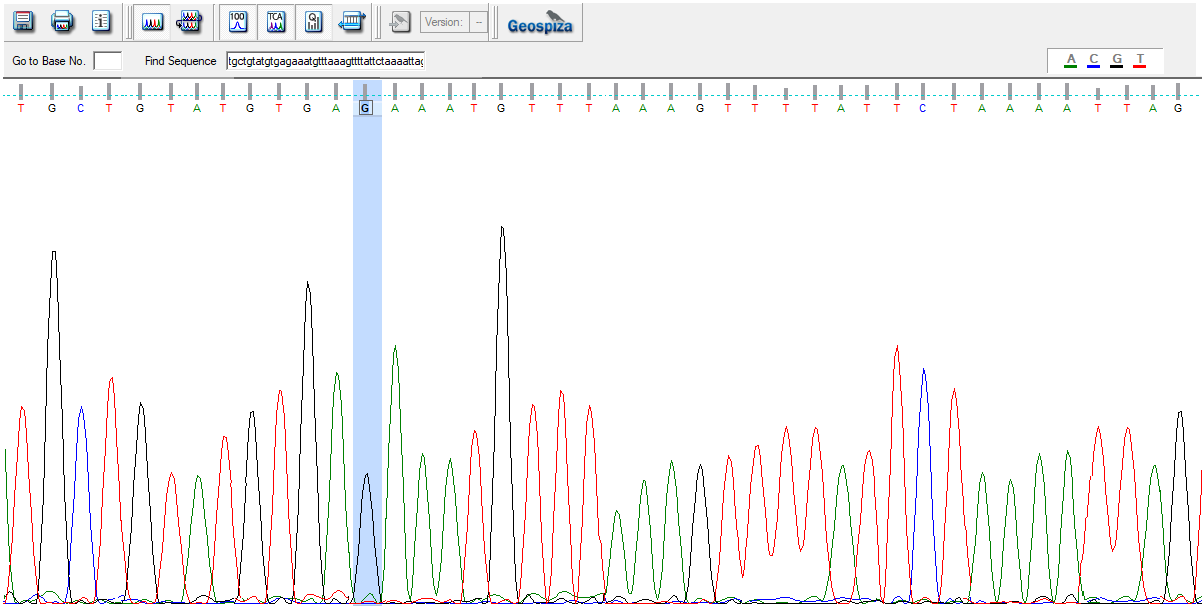


**Fig S4.** Direct DNA Sanger sequencing chromatogram showing a sequence polymorphism (highlighted in blue) in E6 gene region of HPV16 lineage A2 isolate HAM40 (GenBank accession Number: OP572454)


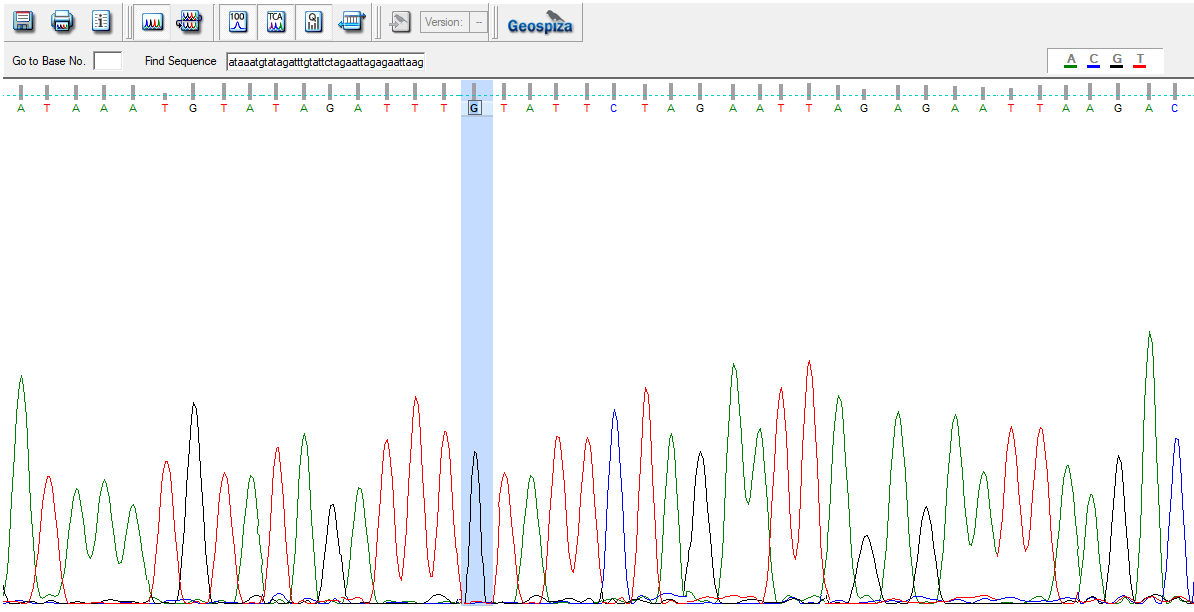


**Fig S5.** Direct DNA Sanger sequencing chromatogram showing a sequence polymorphism (highlighted in blue) in E6 gene region of HPV18 lineage A1 isolate HAM62 (GenBank accession Number: OP572476)


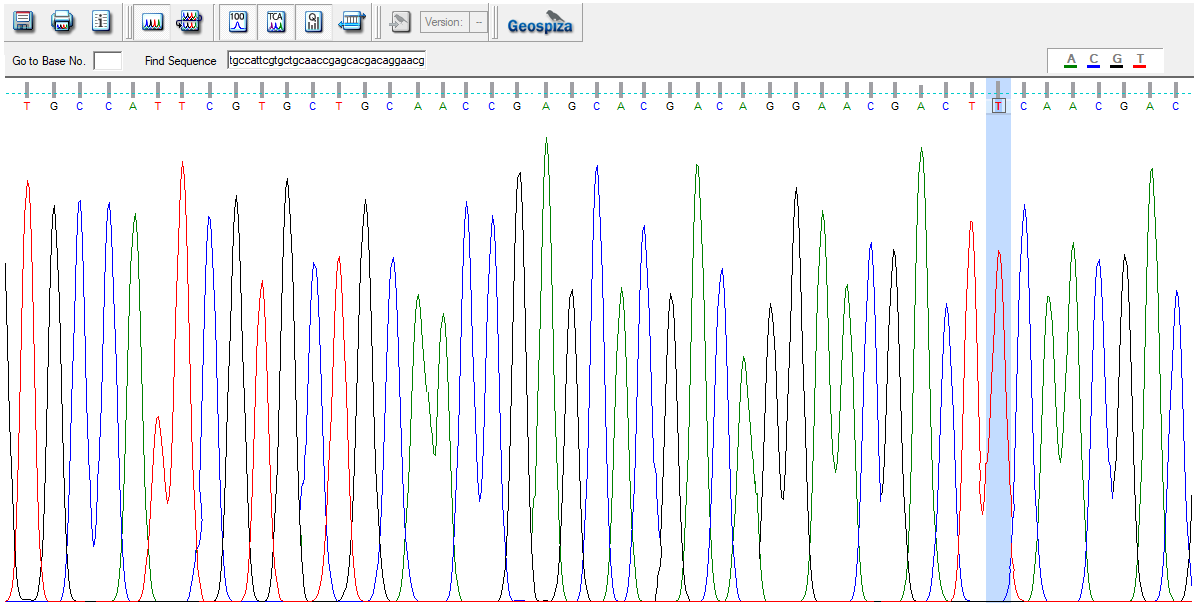


**Fig S6.** Direct DNA Sanger sequencing chromatogram showing a sequence polymorphism (highlighted in blue) in E6 gene region of HPV18 lineage A1 isolate HAM77 (GenBank accession Number: OP572491)


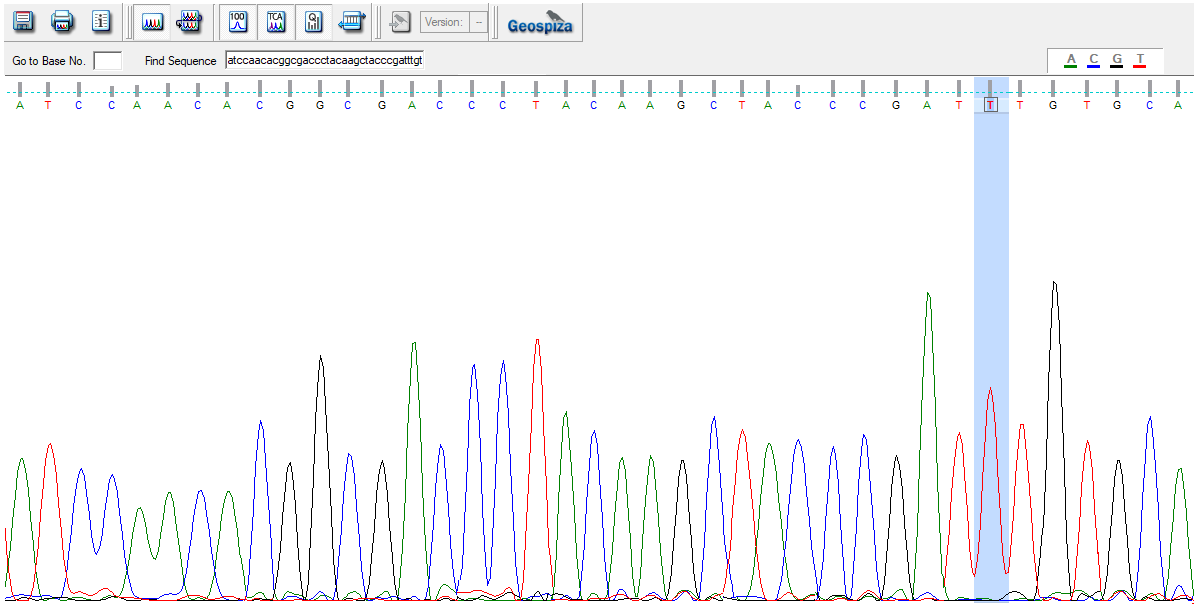


**Fig S7.** Direct DNA Sanger sequencing chromatogram showing a sequence polymorphism (highlighted in blue) in E6 gene region of HPV18 lineage A5 isolate HAM95 (GenBank accession Number: OP572509)
